# Neural Flux-Domain Encoder Resilient to Rotor Eccentricity in BLDC Drives

**DOI:** 10.3390/s26010050

**Published:** 2025-12-20

**Authors:** Hubert Milanowski, Adam K. Piłat

**Affiliations:** Department of Automatic Control and Robotics, Faculty of Electrical Engineering, Automatics, Computer Science and Biomedical Engineering, AGH University of Krakow, A. Mickiewicza 30, 30-059 Krakow, Poland; milan@agh.edu.pl

**Keywords:** data-driven encoder, neural network, magnetic flux density sensing, rotor angle estimation, rotor eccentricity, dynamic eccentricity, low-speed operation, permanent magnet synchronous motor, brushless DC motor

## Abstract

This paper presents a magnetic-flux-based encoder for BLDC drives that maintains high accuracy under rotor eccentricity and dynamic transients. Conventional Hall-sensor-based angle estimators rely on ideal sinusoidal flux assumptions and degrade in the presence of air-gap distortion or misalignment. To overcome these limitations, a nonlinear autoregressive network with exogenous inputs (NARXNet) is proposed as a temporal neural observer that learns the nonlinear, time-dependent mapping between measured flux densities and the true electrical rotor angle. A physics-informed data augmentation framework combines experimentally measured magnetic flux maps with dynamic simulation to generate diverse training scenarios at low and variable speeds. Validation demonstrates mean angular errors below 2°, 95th-percentile errors under 5°, and negligible drift, with enhanced resilience to eccentric displacement and acceleration transients compared to classical methods. The proposed approach provides a compact, data-driven sensing solution for robust, encoderless electric drive control.

## 1. Introduction

Permanent magnet synchronous motors (PMSMs) and brushless direct current (BLDC) machines are widely employed in modern motion control systems owing to their high efficiency, power density, and precise controllability. Accurate rotor position information is essential for high-performance operation, as the stator-generated magnetic field must be properly aligned with the rotor field to maximise torque and minimise losses [1,2,3].

Bearingless motors integrate torque generation and active magnetic suspension within a single electromagnetic structure. In such systems, rotor angle estimation becomes even more critical, as the same angular position feedback governs both torque production and radial force generation for levitation. Position errors may induce cross-coupling between the drive and suspension channels, leading to vibration, reduced efficiency, or even loss of levitation stability [4,5]. Consequently, rotor angle sensing in bearingless drives must achieve higher precision and robustness than in conventional PMSM or BLDC configurations.

Mechanical sensors, such as encoders and resolvers, provide accurate rotor angle feedback but increase cost, volume, and susceptibility to vibration [6,7,8]. Hence, many compact and cost-sensitive drives utilise Hall effect sensors to estimate rotor position from magnetic flux density [9,10]. Digital Hall sensors offer discrete commutation signals but suffer from coarse resolution and interpolation errors at low speeds [11]. Analogue (linear) Hall sensors, by contrast, produce continuous voltage outputs proportional to the rotor’s magnetic field, enabling smoother and more accurate position estimation through trigonometric processing. However, their precision relies on ideal sinusoidal assumptions that often fail under eccentricity, misalignment, or air-gap distortion, resulting in angular ripple and torque fluctuation [12,13,14]. Rotor eccentricity further introduces harmonics into the air-gap flux, with amplitudes and phases that vary with the severity of eccentricity, thereby distorting Hall sensor signals and degrading estimation performance [15,16].

Such non-idealities are common in practical applications, where bearing wear, assembly tolerances, or minor shaft misalignment during motor installation lead to air-gap asymmetry and vibration [17,18,19]. Maintaining accurate position estimation under conditions of eccentricity or sensor offset is therefore crucial for stable operation and torque smoothness [20]. Encoders or sensorless estimation methods robust to eccentricity are thus essential in fault-tolerant and maintenance-limited systems, where mechanical degradation or misalignment cannot be entirely avoided.

Observer-based techniques have long been used to enhance the accuracy and robustness of Hall sensor feedback in electric drives. These approaches model the air-gap field and machine dynamics to infer rotor position from Hall signals, which represent orthogonal components of the air-gap flux density. Classical phase-locked loop (PLL) observers align the Hall sensor vector with the electrical reference [21,22], while extended Kalman filter (EKF) and sliding-mode observer (SMO) variants improve noise immunity and compensate for signal imbalance [23,24,25]. Nonetheless, their accuracy fundamentally depends on the validity of the underlying magnetic model. When eccentricity evolves dynamically with rotation or load, each Hall element experiences time-varying harmonics and phase drift. Under such conditions, observers relying on fixed or weakly nonlinear models lose accuracy as the flux–position mapping becomes nonlinear and history-dependent [26].

To overcome these challenges, data-driven estimators—based on neural networks, support vector regression, or deep learning architectures—seek to learn the nonlinear mapping between distorted Hall voltages and the rotor angle directly from data [27,28]. Such approaches can capture complex magnetic effects, including local saturation and temperature drift, without explicit electromagnetic modelling, achieving high precision even under non-ideal conditions [29]. Hybrid observers combine analytical and data-driven components, applying learned corrections to mitigate model errors while preserving stability and interpretability [30].

Despite these advances, no existing data-driven or hybrid method reliably reconstructs the electrical rotor angle when the air-gap flux is distorted by static or dynamic eccentricity, misalignment, or mechanical tolerances. Under these conditions, the flux–angle relationship becomes nonlinear, time-varying, and history-dependent, undermining the assumptions that classical observers and modern learning-based estimators rely on. The scientific problem addressed in this study is therefore to obtain encoder-grade rotor angle information from minimal Hall sensor measurements when the magnetic field is asymmetric, rich in harmonics, and dynamically modulated by eccentric displacement. Solving this problem requires an estimator capable of learning evolving flux signatures, maintaining stability under transients, and tolerating geometric non-idealities without detailed electromagnetic modelling.

To address these challenges, the present study proposes a nonlinear autoregressive network with exogenous input (NARXNet) as a temporal learning observer for Hall-sensor-based rotor angle estimation. By incorporating delayed inputs and outputs, the NARX structure embeds dynamic memory into the estimation process, enabling it to learn both instantaneous nonlinearities and their temporal evolution. This bridges the gap between rigid model-based observers and memoryless data-driven estimators, facilitating high-fidelity rotor angle estimation and enhanced robustness under varying eccentricity—thus providing a reliable sensing foundation for torque and levitation control in bearingless PMSMs.

## 2. Materials and Methods

### 2.1. Measurement Instrumentation

The flux-domain virtual encoder is implemented on a custom BLDC-like prototype drive. The machine features an external rotor with eight surface-mounted permanent magnet poles (five N38 magnet bars per pole) and a fractional-slot concentrated-winding stator. The stator is equipped with six independently driven coils, each with a measured resistance of 1.21 Ω and an inductance of 592 μH, measured at 10 kHz.

**Figure 1:**
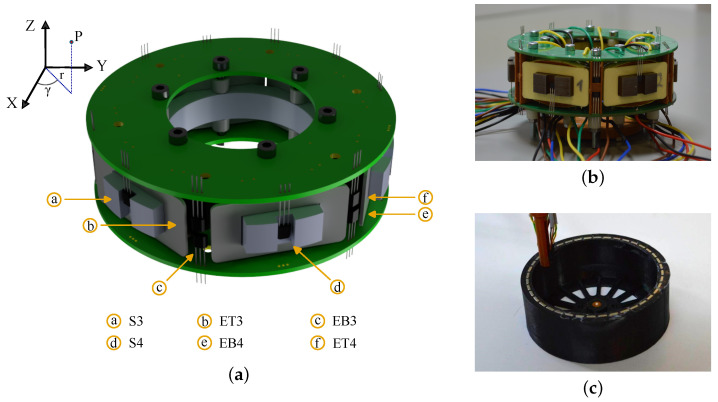
(**a**) Layout of Hall sensor arrangement in the prototype motor; (**b**) stator with mounted Hall sensors on the PCB; and (**c**) rotor of the designed drive system.

Rotor-induced flux density is measured using three linear Hall effect sensors mounted on a PCB embedded in the stator slot region. The sensors are positioned to maximise sensitivity to rotor-generated radial flux while suppressing the stator-generated magnetic field contribution. Each device (Honeywell SS49E) provides a linear ±100 mT operating range with bandwidth exceeding the 1 kHz sampling rate. Symmetric routing, short analogue paths, local shielding, and reproducible sensor spacing (±0.1 mm mechanical tolerance) ensure consistent spatial measurements. A corresponding diagram and table listing the spatial coordinates of ET3–ET5 (Figure 1, Table 1) provide the exact geometric configuration used for data acquisition. A detailed measurement uncertainty analysis is presented in a companion study [31].

The Hall signals are interfaced with a Humusoft real-time I/O board equipped with dedicated ADC channels for each input. Because all channels are sampled in parallel, no multiplexer switching occurs, preserving the timing alignment essential for flux-domain feature extraction.

A one-time offset calibration is performed during initialisation with the rotor removed and the stator unenergised. The intrinsic offset of each sensor is measured and removed; no gain calibration is required. The sensor PCB and acquisition chain therefore provide a compact, low-cost magnetic field measurement subsystem, while the flux-domain virtual encoder reconstructs the electrical angle directly from the measured flux density signatures, including those affected by eccentricity and geometric non-idealities.

### 2.2. Observer Plant Model

The objective of the observer is to estimate the electrical rotor angle, $\theta_{e}\left(t\right)$, from air-gap flux density measurements. The electrical angle relates to the mechanical rotor angle, $\theta_{m}\left(t\right)$, by $\theta_{e}\left(t\right)=p\;\theta_{m}\left(t\right)$, where *p* denotes the number of pairs of motor poles. Three linear Hall sensors, placed with an electrical separation of $\frac{2}{3}\pi$, measure the radial flux density at the stator bore angles $\left\{\varphi_{a},\;\varphi_{b},\;\varphi_{c}\right\}$. The measurement from sensor m∈{a,b,c} is modelled as

(1) $$x_{m}\left(t\right)=B_{r}\left(\varphi_{m},t\right)+b_{m}+\eta_{m}\left(t\right),$$

where $B_{r}\left(\varphi_{m},t\right)$ is the true radial flux density at location $\varphi_{m}$, $b_{m}$ accounts for sensor bias or gain drift, and $\eta_{m}\left(t\right)$ represents measurement noise.

The air-gap flux density distribution, $B_{r}\left(\varphi ,t\right)$, may be represented using a Fourier series expansion. This formulation captures not only the ideal balanced fundamental component but also key non-ideal effects, including spatial harmonics and eccentricity-induced sidebands. Introducing a small eccentricity parameter, ε, to characterise the degree of rotor eccentricity, the flux density at angular position φ can be expressed as follows [32]:

(2) $$H\left(\varphi ,t\right)=B_{0}+\sum_{k=1}^{\infty }A_{k}\sin\;\left(k\theta_{e}\left(t\right)-k\varphi +\psi_{k}\right),$$

(3) $$B_{r}\left(\varphi ,t\right)=H\left(\varphi ,t\right)\;\left[\;1+\varepsilon \;m_{e}\left(\varphi ,t\right)\;\right],$$

where $m_{e}\left(\varphi ,t\right)$ is a zero-mean first-order modulation function accounting for static and dynamic eccentricity. The eccentricity magnitude ε is typically defined as

(4) $$\varepsilon =\frac{\Delta e}{g_{0}},$$

with Δe being the radial offset between the rotor and stator centres and $g_{0}$ the nominal uniform air-gap length. This definition reflects the small eccentricity assumption ε≪1 under which the modulation in (3) remains linear to first order. Physically, eccentricity perturbs the local air gap and thus modulates the ideal flux distribution. Expanding (3) reproduces sidebands around the harmonics in (2). For a detailed analysis, the reader is referred to our companion paper [31].

The three sensor signals, $x_{a}$, $x_{b}$, and $x_{c}$, are obtained by evaluating (3) at the corresponding sensor locations. To facilitate angle estimation, these measurements are processed using the Clarke transform (also known as the αβ0 transform), which maps the three-phase quantities onto two orthogonal axes in the αβ-plane, together with a zero-sequence component. This projection yields a compact representation that isolates the rotational information within the αβ components:

(5) $$\left[\begin{array}{c} x_{\alpha }\\ x_{\beta }\\ x_{0} \end{array}\right]=\frac{2}{3}\left[\begin{array}{ccc} 1 & -\frac{1}{2} & -\frac{1}{2}\\ 0 & \frac{\sqrt{3}}{2} & -\frac{\sqrt{3}}{2}\\ \frac{1}{2} & \frac{1}{2} & \frac{1}{2} \end{array}\right]\left[\begin{array}{c} x_{a}\\ x_{b}\\ x_{c} \end{array}\right].$$

This linear transformation separates the rotational subspace, represented by $\left(x_{\alpha },x_{\beta }\right)$, from the common-mode component $x_{0}$. Although it is possible to operate directly in the abc frame, doing so entangles the phase information with slow drifts and common-mode offsets, thereby forcing the learner to disentangle these effects prior to extracting the rotor angle. In contrast, the αβ0 representation introduces a physics-informed inductive bias: the $\left(x_{\alpha },x_{\beta }\right)$ components form a distorted ellipse with multi-harmonic ripples, while $x_{0}$ aggregates temperature-, supply-, and EMI-induced biases into a single channel.

Under perfectly balanced conditions, the Clarke components satisfy $x_{\alpha }\left(t\right)=A_{1}\cos\theta_{e}\left(t\right),\;$$x_{\beta }\left(t\right)=A_{1}\sin\theta_{e}\left(t\right),x_{0}=0$, which corresponds to a unit circle in the $\left(x_{\alpha },x_{\beta }\right)$ plane after normalisation. In the presence of non-idealities (harmonics, offsets, and eccentricity), the trajectory $\left(x_{\alpha },x_{\beta }\right)$ deforms into a rotated and shifted ellipse with angle-synchronous ripples, while $x_{0}\neq 0$.

To exploit this structure, the αβ0 representation is employed as the observer’s feature space for angle estimation. For stability and identifiability, the observer predicts the unit circle embedding $y\left(t\right)={\left[\sin{\hat{\theta }}_{e}\left(t\right),\;\cos{\hat{\theta }}_{e}\left(t\right)\right]}^{⊤}$ and recovers the estimated angle via ${\hat{\theta }}_{e}\left(t\right)=atan2\;\left(\sin{\hat{\theta }}_{e},\;\cos{\hat{\theta }}_{e}\right)$. This formulation enforces unit amplitude and encourages the model to dewarp ellipticity and suppress offsets prior to the final mapping.

### 2.3. Data Augmentation Pipeline

**Figure 2:**
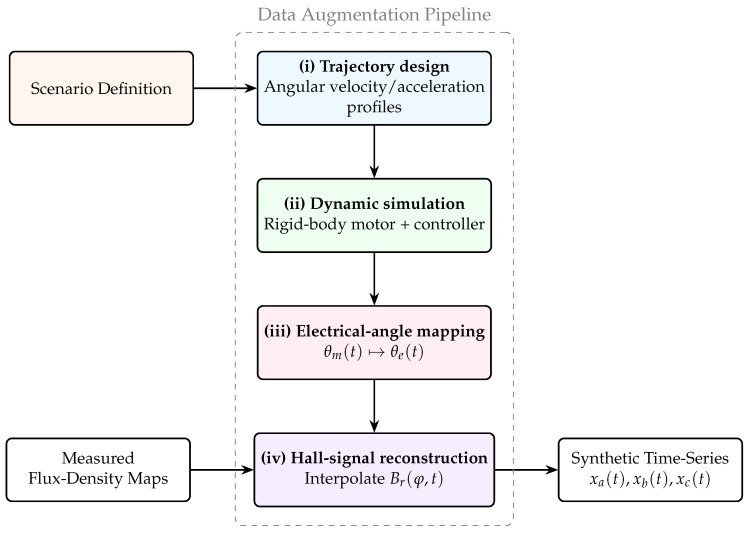
Data augmentation pipeline. Scenario definitions specify rotor motion profiles, which are used by a lightweight rigid-body motor simulation to generate dynamic motion traces. The resulting mechanical angles are converted to electrical angles, and measured flux density maps serve as spatial templates to reconstruct the corresponding Hall sensor signals.

To expose the observer to diverse operating conditions with minimal experimental effort, high-fidelity measurements are combined with physics-informed synthesis. Flux density maps, $B_{r}\left(\varphi \right)$, acquired in situ at multiple eccentricity levels, preserve spatial harmonics and distortions but primarily capture steady or quasi-static behaviour, offering limited transient coverage. To address this limitation, a transformation- and model-based augmentation framework (Figure 2) is introduced to generate realistic time-series signals from static field maps using a simplified rigid-body rotor model and actuator constraints.

The framework exploits the known sensor geometry and identified motor parameters to reinterpret measured spatial fields as time-varying signals under virtual motion. From a set of flux density maps, dynamic scenarios are constructed through trajectory design and kinematic projection, after which the corresponding Hall sensor signals are synthesised from the measured spatial harmonics. This approach enables a dataset that emulates diverse operating conditions without modifying the experimental setup. The underlying in situ scanning framework is documented in full detail in [31].

To maintain physical consistency and reproducibility, the augmentation process is organised into the following stages:

- **(i) Trajectory design.** Rotor speed and acceleration profiles are generated to span near-standstill and the low-speed range of the motor, including constant-speed plateaus, linear acceleration/deceleration ramps, smooth S-curve profiles, and occasional small disturbance injections to emulate load steps. The profiles respect physical limits and the identified test-bench torque–speed characteristic. Rise and fall times, dwell durations, and target values are randomised with a fixed seed for reproducibility. The rotor speed ranges from 0 to 120RPM, and the signals are sampled at Δt=1ms.
- **(ii) Dynamic simulation model.** A lightweight rigid-body bearingless-motor plant is employed to generate dynamically consistent motion profiles and controller interactions. The plant aggregates lumped mass–inertia and linear centring stiffness/damping and is driven by a cascaded motion control structure (outer speed regulation, inner position/force, or current mapping) subject to actuator limits (torque/current saturation). Disturbance channels represent typical low-speed effects, such as unbalance, weak anisotropy, and slow bias drifts. The model outputs {x(t),y(t),ω(t),θ(t)} along with controller traces, yielding dynamically consistent time series. The model equations and parameterisation follow [33].
- **(iii) Electrical angle mapping.** Each mechanical angle trajectory $\theta_{m}\left(t\right)$ is converted to an electrical angle using $\theta_{e}\left(t\right)=p\;\theta_{m}\left(t\right)$. Angle wrapping and unwrapping are handled to avoid discontinuities at 2π, and $\theta_{e}\left(t\right)$ is resampled on a uniform time grid to match the desired sampling rate.
- **(iv) Hall signal reconstruction.** Using pre-recorded spatial flux maps as templates, the expected signals at each Hall sensor along the trajectory from (iii) are synthesised. For each timestamp, the flux density $B_{r}\left(\varphi ,t\right)$ at the sensor azimuths $\left\{\varphi_{a},\;\varphi_{b},\;\varphi_{c}\right\}$ is obtained by a three-dimensional gridded interpolation spline of measured databases $B_{q}\left(x,y,\theta \right)$, ensuring that real spatial harmonics and nonlinearities are preserved as the field evolves under virtual motion. The interpolation is accurate primarily within the bounds of the measurement nodes; outside these nodes, i.e., beyond the scanned spatial or angular range, the approximation may degrade. Therefore, the interpolation is performed relative to the centre of the measured domain, respecting the limits of the recorded data. The resulting signals $x_{a}\left(t\right)$, $x_{b}\left(t\right)$, and $x_{c}\left(t\right)$ retain the spatial content derived from the bench while following the designed kinematics.

**Scope and limitations.** The augmentation focuses on the low-speed regime and does not explicitly model high-frequency effects, such as *PWM* switching ripple, current-dependent magnetic saturation, or strong drive nonlinearities at higher speeds. Consequently, fidelity is highest when eccentricity, offsets, spatial harmonics, and timing skew dominate the error budget, and it may degrade at elevated electrical frequencies. The framework is designed to challenge the observer with distortions and uncertainties most relevant near zero and low speeds—the region most demanding for rotor angle estimation. Readers interested in higher-frequency phenomena are referred to prior work extending the plant model with *PWM* switching ripple, current-dependent saturation, inverter dead time, and frequency-dependent losses; these methods can be incorporated into the present pipeline to improve fidelity at higher speeds [34,35,36].

### 2.4. Baseline Angle Extraction Methods

As a classical baseline for electrical angle estimation, an algebraic Clarke projection combined with a four-quadrant arc-tangent function is employed. Given the components $\left(x_{\alpha }\left(t\right),x_{\beta }\left(t\right)\right)$, the baseline angle is defined as

(6) $$\theta_{e}^{\mathrm{base}}\left(t\right)=atan2\;\left(x_{\beta }\left(t\right),\;x_{\alpha }\left(t\right)\right),$$

which returns an angle, in radians, representing the orientation of the vector in the α–β plane. In an ideally balanced and healthy machine with perfectly aligned sensors, (6) coincides with the true electrical angle up to a constant offset.

To accommodate occasional differences in sign or convention arising from wiring or sensor placement, the swapped variant $atan2\;\left(x_{\alpha }\left(t\right),\;x_{\beta }\left(t\right)\right)$ is also evaluated. The variant yielding the lowest mean absolute error on the validation split is reported as the baseline.

### 2.5. Neural Observer Architecture

**Figure 3:**
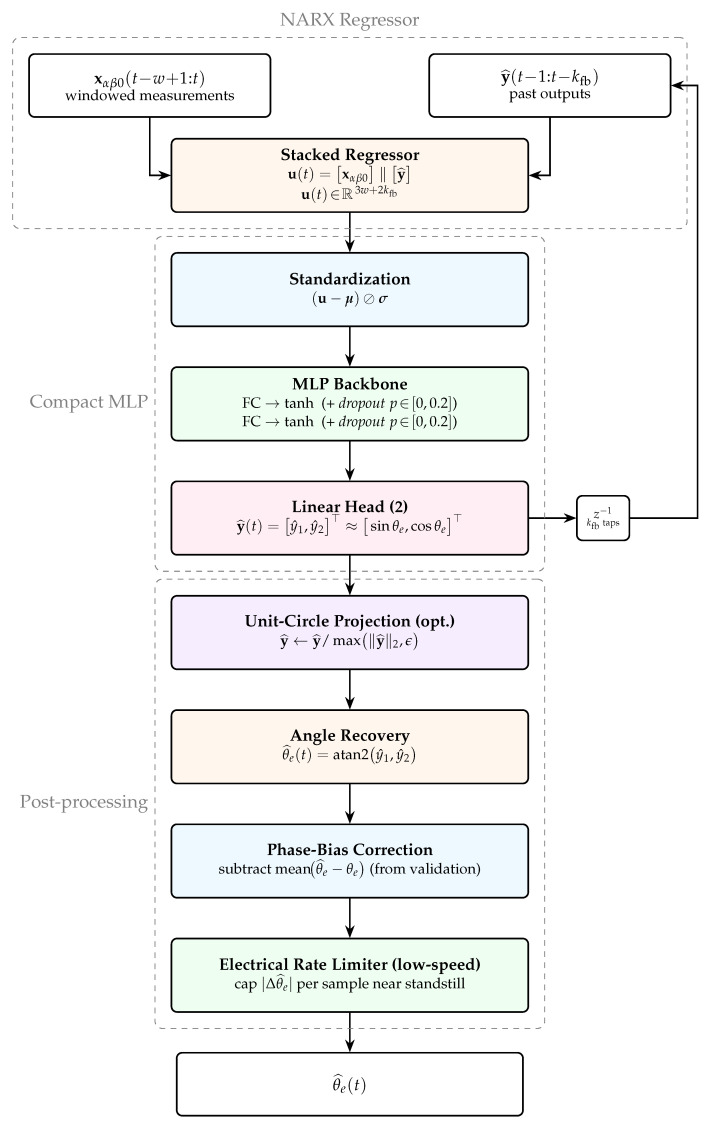
NARX-based neural observer. Windowed αβ0 measurements and recent outputs form the stacked regressor u(t), which is standardised and fed to a two-layer tanh MLP producing $\left(\sin{\hat{\theta }}_{e},\cos{\hat{\theta }}_{e}\right)$. Post-processing applies optional unit circle projection, atan2 recovery, phase bias correction, and a low-speed rate limiter. Dashed boxes highlight the regressor, MLP, and post-processing; the $z^{-1}$ block provides the feedback path.

To estimate the rotor electrical angle in real time from a short history of input features, the observer is implemented as a data-driven model; the general schematic is illustrated in Figure 3. Rather than predicting the scalar angle, ${\hat{\theta }}_{e}\left(t\right)$, directly, the observer estimates its embedding on the unit circle, $y\left(t\right)={\left[\;\sin{\hat{\theta }}_{e}\left(t\right),\;\cos{\hat{\theta }}_{e}\left(t\right)\;\right]}^{⊤}$. This representation is continuous and avoids wrap-around discontinuities. Let the model output be $\hat{y}\left(t\right)={\left[\;{\hat{y}}_{1}\left(t\right),\;{\hat{y}}_{2}\left(t\right)\;\right]}^{⊤}\approx {\left[\;\sin{\hat{\theta }}_{e}\left(t\right),\;\cos{\hat{\theta }}_{e}\left(t\right)\;\right]}^{⊤}$. The estimated angle is then recovered through ${\hat{\theta }}_{e}\left(t\right)=atan2\;\left({\hat{y}}_{1}\left(t\right),\;{\hat{y}}_{2}\left(t\right)\right)$, which preserves continuity across the 2π boundary.

The observer employs a nonlinear autoregressive model with exogenous inputs (NARX), which ingests a window of recent measurements together with a short history of its own past outputs to produce the current estimate. Let $x_{\alpha \beta 0}\left(t\right)={\left[\;x_{\alpha }\left(t\right),\;x_{\beta }\left(t\right),\;x_{0}\left(t\right)\;\right]}^{⊤}$. For window length *w* and feedback depth $k_{\mathrm{fb}}$, the stacked regressor is $u\left(t\right)=\left[\;x_{\alpha \beta 0}\left(t\;-\;w\;+\;1:t\right)\;\right]\;∥\;\left[\;y(t\;-\;k_{\mathrm{fb}}:t\;-\;1)\;\right]\in R^{\;3w+2k_{\mathrm{fb}}}$, where ‖ denotes concatenation and the slice notation lists samples from oldest to most recent. This construction preserves phase continuity and direction while enabling inference from the recent αβ0 trajectory.

The mapping $y\left(t\right)=f_{\Theta }\;\left(u(t)\right)$ is realised by a compact, fully connected, feedforward multilayer perceptron (MLP) with two hidden layers, which is sufficient for low-dimensional input. The main components are as follows:

- **Backbone.** Two tanh hidden layers with modest widths, selected through hyperparameter tuning. Optional dropout after each hidden layer (p∈[0,0.2]) provides regularisation and mitigates overfitting.
- **Output layer.** A two-unit linear head producing $\left(\sin{\hat{\theta }}_{e},\;\cos{\hat{\theta }}_{e}\right)$. During inference, the output is optionally projected onto the unit circle to enforce $\sin^{2}\theta +\cos^{2}\theta =1$:
  $$\hat{y}\left(t\right)\;\leftarrow \;\frac{\hat{y}\left(t\right)}{\max\;\left({∥\hat{y}\left(t\right)∥}_{2},\;\epsilon \right)},\;\epsilon >0.$$
- **Normalisation.** Inputs are standardised feature-wise using training set statistics (mean μ and standard deviation σ), which are held fixed during validation and testing.

To enhance robustness during inference, the following stabilisation mechanisms are applied:

- **Unit circle projection** suppresses amplitude drift in ${∥\hat{y}\left(t\right)∥}_{2}$ and reduces angular bias.
- **Phase bias correction** subtracts a constant offset—estimated on the validation set as the mean of ${\hat{\theta }}_{e}\left(t\right)-\theta_{e}^{\mathrm{true}}\left(t\right)$—during operation.
- **Electrical rate limiter** constrains the per-sample increment $|\Delta {\hat{\theta }}_{e}|$ near standstill to values consistent with motor speed and acceleration limits, rejecting implausible spikes.

### 2.6. Training Procedure

Learning proceeds in two phases to stabilise the feedback loop. In **Phase 1 (teacher forcing),** the network is trained in a purely supervised manner using ground-truth feedback. For each time instant *t*, the NARX input stacks a window of Clarke features together with the *true* past outputs, $u\left(t\right)=\left[\;x_{\alpha \beta 0}\left(t\;-\;w\;+\;1:t\right)\;\right]\;∥\;\left[\;y_{\mathrm{true}}\left(t\;-\;1:t\;-\;k_{\mathrm{fb}}\right)\;\right]$, so that the model learns a one-step prediction under perfect feedback. Training continues until the mean-squared error (MSE) plateaus.

In **Phase 2 (closed-loop fine-tuning with scheduled sampling),** the model is exposed to its own, potentially imperfect, feedback. After seeding the first *F* steps with ground truth, the feedback signal at time *t* is selected probabilistically: with probability $P_{\mathrm{model}}$ the previous model output $\hat{y}\left(t\;-\;1\right)$ is used, and with complementary probability $1-P_{\mathrm{model}}$ the true value $y_{\mathrm{true}}\left(t\;-\;1\right)$ is used. The mixing probability is gradually increased during fine-tuning (e.g., $P_{\mathrm{model}}:0.1\to 0.9$) so that later epochs emulate deployment conditions.

During Phase 2, **jitter noise** may be injected into the feedback angle prior to conversion to its (sin,cos) embedding: with probability $P_{\mathrm{noise}}\in \left\{0.25,0.5\right\}$, a perturbation $\delta \theta ∼N(0,\sigma^{2})$ with σ= 3° is added. This promotes robustness against small defects in past estimates. Optionally, **label smoothing** applies a short moving average to the target sequence with window length $N_{\mathrm{smooth}}\in \left\{5,9\right\}$, followed by re-projection onto the unit circle.

The training objective is defined as $L={∥\hat{y}\left(t\right)-y_{\mathrm{true}}\left(t\right)∥}_{2}^{2}$, which is minimised using the Adam optimiser with a learning rate of $10^{-3}$. Training is performed on mini-batches of sequential data, typically for 20 epochs in Phase 1 and an additional 10 epochs in Phase 2.

### 2.7. Evaluation Metrics

Accuracy and robustness are evaluated by comparing the estimated rotor angle ${\hat{\theta }}_{e}\left(t\right)$ with the encoder reference $\theta_{e}^{\mathrm{true}}\left(t\right)$. Angle errors are wrapped onto the principal branch to avoid 2π discontinuities:

$$\Delta \theta_{e}\left(t\right)={\mathrm{wrap}}_{\left(-\pi ,\pi \right]}\;\;\left({\hat{\theta }}_{e}\left(t\right)-\theta_{e}^{\mathrm{true}}\left(t\right)\right).$$

Unless otherwise stated, angles are reported in degrees (°) for interpretability, although computations are performed in radians.

- **Mean Absolute Error (MAE):** $\frac{1}{N}\sum_{t=1}^{N}|\Delta \theta_{e}\left(t\right)|$.
- **Root Mean Square Error (RMSE):** $\sqrt{\frac{1}{N}\sum_{t=1}^{N}{\left(\Delta \theta_{e}\left(t\right)\right)}^{2}}$.
- **95th-Percentile Error (P95):** The 95th percentile of $\left|\Delta \theta_{e}\left(t\right)\right|$, indicating a typical worst-case error under nominal conditions.
- **Circular bias (mean signed error):** $\frac{1}{N}\sum_{t=1}^{N}\Delta \theta_{e}\left(t\right)$, capturing systematic lead or lag.
- **Outlier rate:** $\frac{1}{N}\sum_{t=1}^{N}1\;\left(|\Delta \theta_{e}\left(t\right)|>\theta_{\mathrm{thr}}\right)$, with $\theta_{\mathrm{thr}}=$ 5° unless otherwise indicated.
- **Drift (low-frequency trend):** Compute a low-frequency trend $\Delta \theta_{e}^{\mathrm{LF}}\left(t\right)$ using a 1.5s moving average of $\Delta \theta_{e}\left(t\right)$, and then report the slope (deg/s) of a least-squares linear fit over time; values near zero indicate good long-term calibration.

### 2.8. Hyperparameter Tuning

The objective is to achieve strong *closed-loop* (CL) accuracy in the low-speed regime while satisfying real-time constraints. Models are trained on a CPU within a fixed computational budget, employing the Adam optimiser, early stopping, and gradient-norm clipping. Each configuration is evaluated using three random seeds, with the final selection based on the *median* RMSE of the CL validation set. When multiple configurations exhibit a comparable median CL validation RMSE, the preferred model is chosen according to the following criteria, in order of priority: (i) lower P95, (ii) lower RMSE, (iii) lower outlier rate, and (iv) lower inference latency. The discrete search grid explored during hyperparameter tuning is summarised in Table 2.

## 3. Results and Discussion

After approximately 1300 hyperparameter-tuning trials, the selected configuration—window depth w=4; feedback depth $k_{\mathrm{fb}}=2$; hidden widths [32,16]; dropout =0.20; scheduled sampling $p_{\mathrm{model}}=0.90$; feedback jitter $p_{\mathrm{noise}}=0.50,\;\sigma =0$; and label-smoothing window $n_{\mathrm{smooth}}=0$—achieved a closed-loop validation RMSE of 2.30° (P95 4.45°, MAE 1.87°, bias −0.50°, drift 0.03°, and outlier rate 2.48%) and is used as the reference configuration in the subsequent discussion.

### 3.1. Quantitative Estimation Accuracy

**Figure 4:**
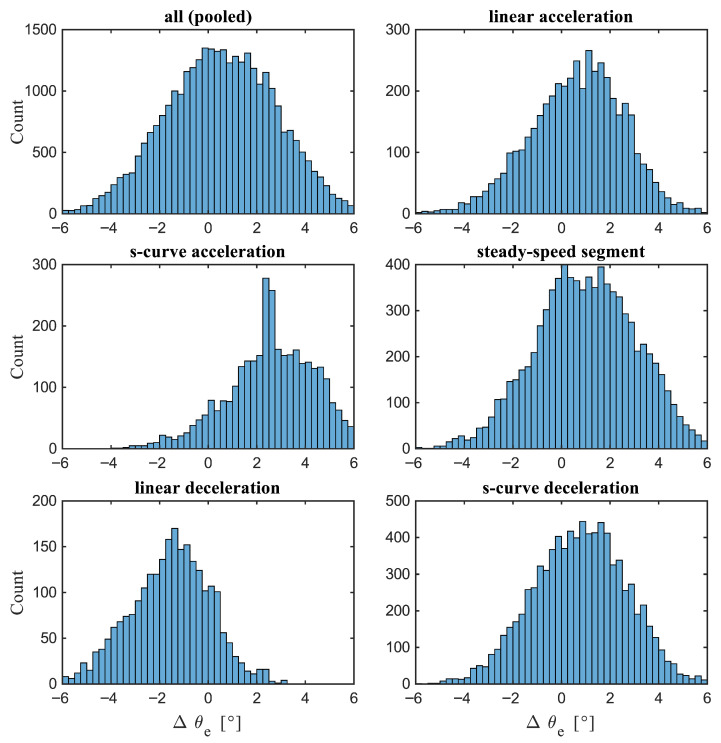
Histogram of angular position estimation error ($\Delta \theta_{e}$) for different motion phases. Distributions are shown for the full trajectory (pooled) and for individual segments: linear acceleration, S-curve acceleration, steady-speed motion, linear deceleration, and S-curve deceleration. Errors are centred near zero for all phases, indicating generally unbiased estimates. Steady-speed segments exhibit a tight, symmetric distribution around zero, demonstrating high accuracy under constant-velocity operation.

Figure 4 illustrates the distribution of rotor angle estimation errors for each phase of operation, while Table 3 summarises the corresponding error metrics (MAE, RMSE, 95th percentile (P95), bias, outlier rate, and drift). Overall, errors are tightly clustered around zero, exhibiting near-zero mean bias, modest spread (RMSE on the order of a few degrees), and 95% of values within a few degrees. Deviations rarely exceed approximately 5°, and large errors are uncommon, with outlier rates of only a few percent. The slight tails in the pooled histogram arise primarily from transient phases (acceleration and deceleration), but their low frequency indicates stable behaviour with no runaway error. Drift over the full cycle is effectively zero, as transient offsets are corrected by the end of the cycle, supporting long-term closed-loop stability.

Steady-speed operation yields the best results. At constant velocity, the estimator fully converges and is minimally affected by disturbances or model mismatch. The histogram is sharply peaked at zero, with MAE and RMSE at their lowest values (often well below 1°) and P95 around 1–2°. Bias is essentially zero, outliers are virtually absent, and no drift is observed. From a control perspective, this provides reliable rotor angle feedback, near-optimal torque production, and minimal torque ripple, yielding sensor-like performance in steady state.

During deceleration (both linear and S-curve ramp-down), behaviour mirrors acceleration but with the opposite sign: the estimator tends to lead, producing a small negative bias. The spread is comparable to that during acceleration, with elevated but controlled RMSE and MAE. The histogram exhibits a left tail. S-curve profiles again help contain extremes, whereas abrupt linear segments contribute a few additional negative outliers. Outlier rates remain low, similar to those observed during acceleration, and drift is minimal, as the estimator realigns quickly at the new lower speed or stops.

Breaking down performance by phase reveals predictable trends. During acceleration (both linear and S-curve ramps), the error distribution widens and skews, yielding the highest per-phase RMSE and a small positive bias (phase lag under increasing speed). The histogram exhibits a longer right tail and elevated P95. These are the largest errors observed, yet they remain bounded, with worst cases around 5–8° and 95% of errors below approximately 5°. Outliers are concentrated in this phase but remain rare, typically constituting a single-digit percentage of the acceleration period. Drift during acceleration is small (∼1°), indicating a temporary offset rather than cumulative divergence. Profile smoothness also affects performance: S-curve acceleration noticeably tightens the distribution relative to a linear ramp, as reduced jerk limits transient errors and prevents large spikes.

In summary, accuracy remains high across all operating regimes: nearly ideal during steady state and only modestly reduced during transients. Rapid linear accelerations produce the largest spreads and a small positive bias, while decelerations exhibit the symmetric effect. Smoother, low-jerk profiles consistently reduce error magnitudes and outliers. Even under worst-case conditions, errors remain within a few degrees, with no evidence of divergence or uncontrolled drift. This level of performance is sufficient for real-time motor control: small transient biases (a few degrees) are unlikely to destabilise field-oriented control and can be compensated for, while the near-zero steady-state error ensures efficient and smooth operation.

**Figure 5:**
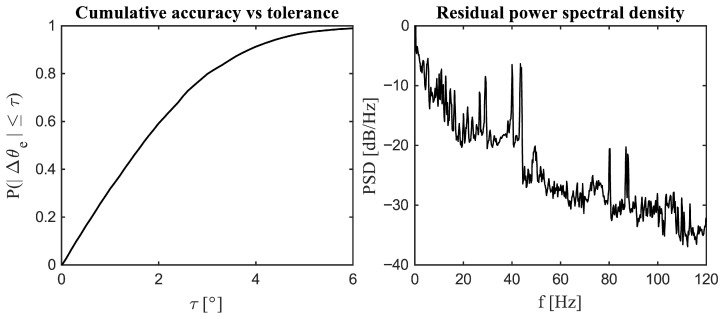
Cumulative accuracy and spectral content of the estimation error. (**Left**): Empirical CDF of the absolute rotor angle error, $F\left(\tau \right)=P(|\Delta \theta_{e}|\leq \tau )$. (**Right**): Power spectral density (PSD) of the zero-mean residual $\Delta \theta_{e}$ (dB/Hz). Together, the plots indicate how frequently a given accuracy threshold is met and the frequency distribution of the residual energy.

Figure 5 summarises these results: the left panel plots the cumulative accuracy curve $F\left(\tau \right)=\mathrm{Pr}(|\Delta \theta_{e}|\leq \tau )$, while the right panel shows the power spectral density (PSD) of the residual $\Delta \theta_{e}\left(t\right)$.

The cumulative curve quantifies accuracy as a function of tolerance. It rises steeply for small τ, indicating that a large fraction of samples lie close to zero error, and then increases more gradually, approaching unity as τ→6°. Consistent with the phase-wise statistics, roughly two thirds of samples satisfy $|\Delta \theta_{e}|\;\leq$ 2°, about 90% satisfy $|\Delta \theta_{e}|\;\leq 4–$4.5°, and at least 95% satisfy $|\Delta \theta_{e}|\;\leq$ 5°, in agreement with the pooled P95 = 4.453° reported in Table 3. This presentation complements the histograms by expressing precision directly as the probability of compliance for any chosen tolerance; for instance, a 5° commutation tolerance corresponds to ≥95% compliance throughout the cycle.

The PSD panel demonstrates that residual energy is concentrated at low frequencies, with a decaying broadband tail at higher frequencies and a few narrowband components. This spectral pattern is typical of slowly varying, transient mismatch (low-frequency bias or phase lag during ramps) with smaller high-frequency contributions. The low-frequency dominance aligns with the phase-dependent bias observed earlier (lag during acceleration and lead during linear deceleration), whereas the minimal high-frequency energy indicates limited disturbance-induced angle jitter. Overall, these results confirm compliance with practical angle tolerances and show that the remaining error is slow and bounded, compatible with closed-loop operation, and amenable to simple filtering or bias compensation strategies.

### 3.2. Qualitative Estimation Accuracy

**Figure 6:**
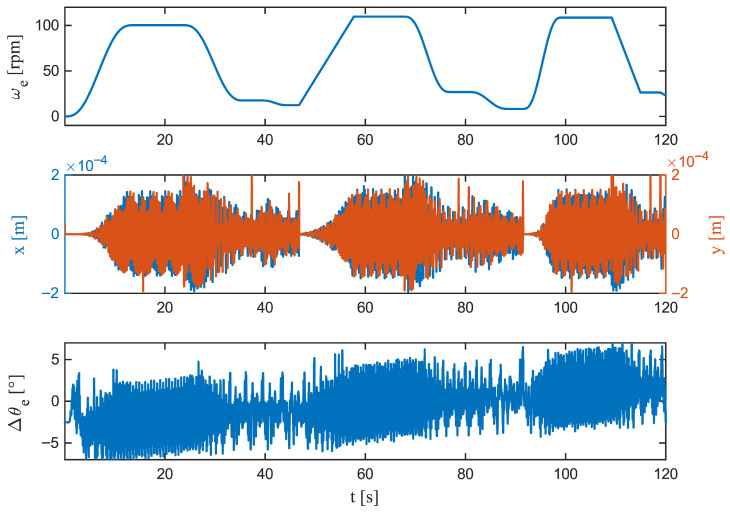
Time-domain overview of the full run. (**Top**): Angular velocity $\omega_{e}\left(t\right)$. (**Middle**): Lateral displacements x(t) and y(t). (**Bottom**): Rotor angle residual $\Delta \theta_{e}={\hat{\theta }}_{e}\left(t\right)-\theta_{e}^{\mathrm{true}}\left(t\right)$. *x*–*y* variations correspond to the very low-frequency component of $\Delta \theta_{e}$, indicating a quasi-static bias rather than broadband jitter.

**Figure 7:**
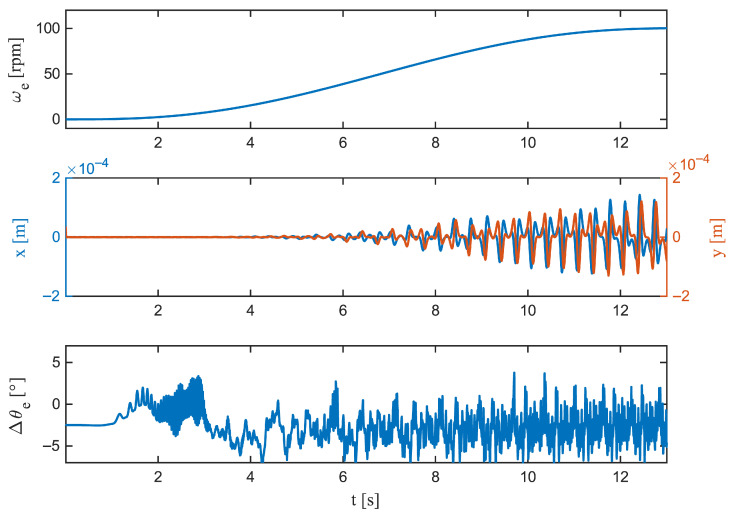
S-curve acceleration (zoom). (**Top**): Angular velocity ω(t), showing the jerk-limited ramp. (**Middle**): Lateral displacements x(t) and y(t). (**Bottom**): Rotor angle residual $\Delta \theta_{e}\left(t\right)={\hat{\theta }}_{e}\left(t\right)-\theta_{e}^{\mathrm{true}}\left(t\right)$. The jerk-limited motion produces a compact transient with a reduced peak $|\Delta \theta_{e}|$, short settling, and a tight spread around zero within ±5°. Any *x*–*y* offset manifests mainly as a mild, slowly varying bias, consistent with low-frequency sensitivity during gradual acceleration.

**Figure 8:**
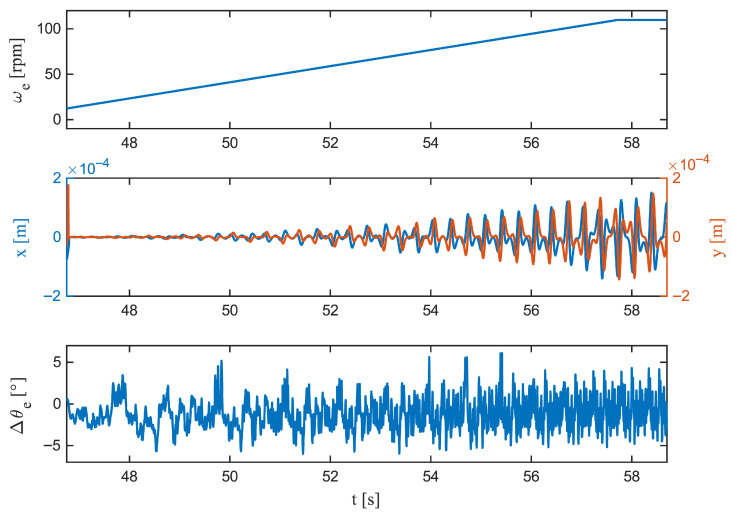
Linear acceleration (zoom). (**Top**): Angular velocity $\omega_{e}\left(t\right)$, showing the constant acceleration ramp. (**Middle**): Lateral displacements x(t) and y(t). (**Bottom**): Rotor angle residual $\Delta \theta_{e}\left(t\right)={\hat{\theta }}_{e}\left(t\right)-\theta_{e}^{\mathrm{true}}\left(t\right)$. The constant acceleration produces the largest transient, with peaks approaching ±5° followed by rapid re-convergence to zero.

**Figure 9:**
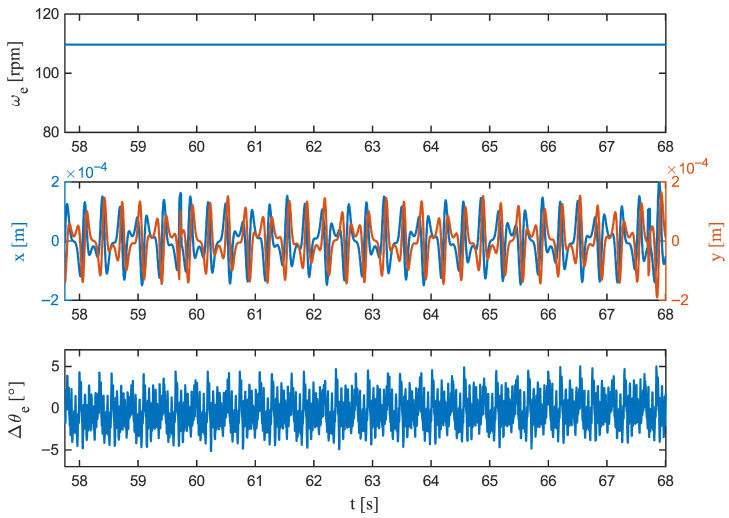
Constant-speed segment (zoom). (**Top**): Angular velocity $\omega_{e}\left(t\right)$, held steady. (**Middle**): Lateral displacements x(t) and y(t), nearly constant. (**Bottom**): Rotor angle residual $\Delta \theta_{e}\left(t\right)={\hat{\theta }}_{e}\left(t\right)-\theta_{e}^{\mathrm{true}}\left(t\right)$. The residual is tightly centred with minimal variance and no outliers relative to the ±5° band, illustrating near-sensor behaviour under nominal conditions.

Figure 6 (whole trajectory), Figure 7 (S-curve acceleration), Figure 8 (linear acceleration), and Figure 9 (steady speed) depict the evolution of the residual $\Delta \theta_{e}\left(t\right)$ under various operating conditions. The summary in Table 4 reinforces the main visual impressions with six compact time-domain indicators. Over the full cycle, the error remains bounded and quickly returns to the ±5° band after transitions. The largest deviation is $|\Delta \theta_{e}{|}_{\mathrm{pk}}=$ 7.86° at $T_{\mathrm{peak}}=6272\;\mathrm{ms}$, the maximum local rate of change reaches 750.12deg/s, and the residual reduces by half within 32ms. Even during this single worst-case excursion, the estimate remains within tolerance for 98.67% of the time, and any continuous exceedance lasts only 92ms, consistent with the brief spikes observed at phase boundaries in Figure 6.

Zooming in on the S-curve acceleration (Figure 7) shows that jerk-limited motion keeps transients compact: the peak deviation is $|\Delta \theta_{e}{|}_{\mathrm{pk}}=$6.15°, occurring early at 5000ms; the growth is comparatively smooth (563.03deg/s), and the decay is rapid ($t_{1/2}=24\;\mathrm{ms}$). Compliance with the ±5° estimation band is virtually perfect ($C_{5{}^{\circ}}=99.89\%$). Linear acceleration (Figure 8) produces a slightly larger peak, $|\Delta \theta_{e}{|}_{\mathrm{pk}}=$ 6.09° at 8660ms, with a sharper rise (727.39deg/s) but an even faster collapse to half amplitude ($t_{1/2}=8\;\mathrm{ms}$). Time within tolerance remains high ($C_{5{}^{\circ}}=99.55\%$) and the longest continuous exceedance is brief (20ms). At steady speed (Figure 9), the residual is tightly centred; the largest instantaneous deviation is $|\Delta \theta_{e}{|}_{\mathrm{pk}}=5.20{}^{\circ}$, occurring late in the window (11,240 ms), the local dynamics are the gentlest among the cases (620.73deg/s), and the error halves within 8ms. The band is maintained for the entire duration ($C_{5{}^{\circ}}=100\%$).

Taken together, the figures and metrics indicate that the observer’s most demanding regime is acceleration, where peaks are largest and changes are sharpest, but deviations beyond ±5° are rare and very brief (4–20 ms in zoomed views). The S-curve motion mitigates both the growth rate and the duration of excursions, while steady operation yields near-sensor behaviour. These patterns align with the distributional results and confirm that the dynamic error is a short-lived transient rather than a sustained drift. Such short and bounded error excursions ensure that the estimator can be integrated into real-time commutation or FOC loops without introducing torque ripple or violating stability margins.

### 3.3. Robustness to Eccentric Displacement Disturbance

To evaluate whether eccentric rotor displacement significantly affects the residual angle, the error signal was low-pass filtered at $f_{c}=16$ Hz to extract the slow component $\Delta \theta_{e}^{\mathrm{LF}}\left(t\right)$, which is most sensitive to geometric influences. Within sliding windows of 0.15 s with 50% overlap, the Pearson correlation coefficients $\rho_{x}$ and $\rho_{y}$ were computed between $\Delta \theta_{e}^{\mathrm{LF}}\left(t\right)$ and the measured displacements x(t) and y(t). The delay corresponding to the absolute peak of the cross-correlation (within ±200 ms) was also extracted. Median values across all windows, together with interquartile ranges, are summarised in Table 5.

The results indicate a negligible systematic coupling between lateral displacement and the low-frequency component of the residual error. Correlations along both axes are centred near zero, with interquartile ranges spanning positive and negative values, while the estimated lags cluster around zero but exhibit broad dispersion. These observations are consistent with a weak, quasi-static effect rather than a time-locked influence. This near-zero coupling confirms that realistic mechanical displacement introduces only a small quasi-static bias and does not meaningfully affect the estimator’s accuracy. When considered alongside phase-resolved time-domain indicators, the findings support a robustness interpretation: realistic eccentric displacements primarily introduce a small, very low-frequency offset that neither accumulates nor generates significant out-of-band excursions.

### 3.4. Baseline Comparisons

**Figure 10:**
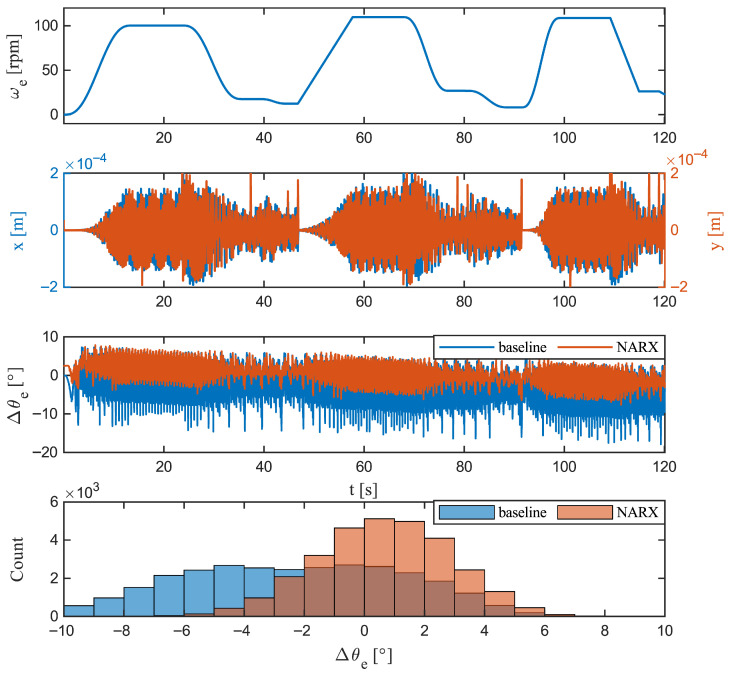
Baseline comparison over the full trajectory. (**Top**): Angular velocity $\omega_{e}\left(t\right)$. (**Middle**): Lateral displacements x(t) and y(t). (**Lower middle**): Rotor angle residual $\Delta \theta_{e}$ for the baseline atan2 observer (blue) and the proposed NARX estimator (orange). (**Bottom**): Pooled histograms of $\Delta \theta_{e}$ for both methods. The baseline exhibits a negative bias with pronounced high-frequency jitter, whereas the NARX residual is narrower, with reduced jitter and a small positive bias.

Figure 10 contrasts the classical αβ-vector method with the NARX estimator over the same drive cycle. The upper panel delineates the sequence of operating regimes, while the displacement traces below show x(t) and y(t) variations, including occasional narrow events. Against this backdrop, the residuals reveal the essential difference between the observers: the baseline exhibits a persistent negative offset that gradually deepens throughout the run and is superimposed on pronounced high-frequency ripple following displacement disturbances; by contrast, the NARX residual is visibly tighter, with substantially less jitter and a small, nearly constant positive offset. These characteristics are reflected in the pooled histograms in the bottom panel: the baseline distribution is left-shifted with heavier tails, whereas NARX is narrower and centred closer to zero.

This behaviour is consistent with the underlying mechanisms of each approach. The baseline is sensitive to cross-coupling, amplitude and phase errors, and geometric imperfections such as lateral displacement (*x*–*y*) or mild eccentricity. Such perturbations distort the current locus and introduce a quasi-static bias together with sideband ripple, which manifests as a broader spread in the baseline trace. In contrast, NARX exploits temporal context; the sequential information allows it to discount momentary waveform distortions and learn an invariant mapping that suppresses high-frequency content and compresses the error envelope. In the presence of *x*–*y* disturbances, the NARX residual exhibits primarily a gentle envelope of low-frequency peaks rather than amplified excursions, indicating robustness to the lateral misalignment and eccentricity effects that typically affect the baseline method.

From a control standpoint, the implications are immediate. The smoother NARX residual reduces torque ripple injection and permits higher cutoff frequencies in additional filtering while preserving transient responsiveness. By contrast, the combination of bias and jitter in the baseline would propagate as a phase error or necessitate aggressive filtering, trading accuracy for latency. Overall, the figure demonstrates that NARX provides a more stable and robust angle estimate throughout the trajectory, particularly under *x*–*y* perturbations and during speed transitions.

## 4. Conclusions

This study presents a data-driven magnetic virtual encoder for rotor angle estimation in electric drives operating under conditions of rotor eccentricity and rapid transients. Central to the approach is a nonlinear autoregressive network with exogenous input (NARXNet), which effectively learns the temporal and nonlinear mapping between distorted Hall sensor signals and the true rotor angle, capturing dynamic eccentricity effects that elude conventional observers. In doing so, the proposed observer bridges the long-standing gap between rigid analytical models and static data-driven estimators by embedding short-term memory and feedback into the learning process.

Compared with conventional encoderless schemes, the proposed flux-domain encoder functions as a compact measurement instrument that transforms sparse magnetic field observations into stable, high-fidelity angle information. Its demonstrated tolerance to geometric imperfections—including both static and dynamic eccentricity—highlights the resilience of the sensing architecture and the advantages of flux-based instrumentation. These characteristics make the approach well suited to applications requiring compact, low-cost, and encoder-independent position-sensing hardware.

Although the results demonstrate promising accuracy and resilience, it should be noted that the present evaluation is limited to low-speed operation, with high-speed performance left for future investigation. Future efforts should include deployment on real-time embedded hardware and evaluation of closed-loop behaviour when integrated with the control algorithm of a full electric drive. This will assess responsiveness and robustness under real electrical noise, inverter nonlinearities, and torque disturbance scenarios. Beyond this, the observer could be extended to high-speed regimes through incorporation of inverter-aware effects such as PWM ripple, current-induced saturation, and frequency-dependent flux distortion. Through these avenues, the proposed flux-based encoder may evolve into a core component of robust, sensorless, and intelligent electric drive systems.

## Figures and Tables

**Table 1:** Arrangement of Hall sensors in cylindrical coordinates.

Sensor Type	Radius *r* [mm]	Angle γ [°]	Height *z* [mm]
ET1…6 (Top)	47.5	$\phi_{i}=60{}^{\circ}\cdot \left(i-1\right),\;i=1\ldots 6$	+5
EB1…6 (Bottom)	47.5	$\phi_{i}=60{}^{\circ}\cdot \left(i-1\right),\;i=1\ldots 6$	−5
S1…6 (Side)	47.5	$\phi_{i}=30+60{}^{\circ}\cdot \left(i-1\right),\;i=1\ldots 6$	$z_{i}=\left\{-5,\;0,\;5,\;-5,\;0,\;5\right\}$

**Table 2:** Grid of tuned hyperparameters used for model selection.

Hyperparameter	Values
Window length *w*	{4,8,12}
Feedback depth $k_{fb}$	{2,4,6}
Hidden widths (layers 1–2)	{(16,8),(32,16)}
Activation function	tanh
Dropout per hidden layer	{0.05,0.20}
Scheduled sampling end $p_{\mathrm{model}}$	{0.1,0.5,0.9} (linear ramp in Phase 2)
Feedback jitter prob. $p_{\mathrm{noise}}$	{0,0.25,0.5}
Feedback jitter std. σ	{0°,3°}
Label smoothing window $n_{\mathrm{smooth}}$	{0,5,9} (1 ms/sample)
Optimiser/LR	Adam/$10^{-3}$
Epochs	Phase 1: ∼20; Phase 2: ∼10
Batching	Sequential; one scenario per batch
Early stopping/clipping	Enabled; gradient-norm clipping

**Table 3:** Per-phase test set errors for NARX rotor angle estimates (see Section 2.7 for metrics).

Scenario Type	MAE	RMSE	P95	Bias	Outlier	Drift
all (pooled)	1.868	2.308	4.453	0.519	0.025	−0.039
linear acceleration	1.634	2.004	3.805	0.618	0.010	−0.142
s-curve acceleration	2.827	3.233	5.485	2.656	0.090	0.084
steady-speed	1.855	2.304	4.432	1.002	0.023	−0.112
linear deceleration	1.857	2.318	4.484	−1.594	0.030	−0.016
s-curve deceleration	1.653	2.036	3.894	−0.739	0.009	−0.086

**Table 4:** Time-domain tracking metrics. The residual is $\Delta \theta_{e}\left(t\right)={\hat{\theta }}_{e}\left(t\right)-\theta_{e}^{\mathrm{true}}\left(t\right)$ in degrees. Definitions: $|\Delta \theta_{e}{|}_{\mathrm{pk}}$—peak absolute residual [deg]; $T_{\mathrm{peak}}$—time from the window/phase start to $|\Delta \theta_{e}{|}_{\mathrm{pk}}$ [ms]; $t_{1/2}$—time from the peak until $\left|\Delta \theta_{e}\right|$ first falls to $\max\;\left(0.5\;|\Delta \theta_{e}{|}_{\mathrm{pk}},\;2{}^{\circ}\right)$ [ms]; ${\left|d\Delta \theta_{e}/dt\right|}_{\mathrm{pk}}$—peak absolute slope of the residual [deg/s]; $C_{5{}^{\circ}}$—time fraction with $|\Delta \theta_{e}|\leq 5{}^{\circ}$ [%]; and $L_{5{}^{\circ}}$—longest continuous interval with $|\Delta \theta_{e}|>5{}^{\circ}$ [ms].

Metric	Whole Run	S-Curve Accel	Linear Accel	Steady Speed
$|\Delta \theta_{e}{|}_{\mathrm{pk}}$	7.86	6.15	6.09	5.20
$T_{\mathrm{peak}}$	6272	5000	8660	11,240
$t_{1/2}$	32	24	8	8
${\left|\frac{d\;\Delta \theta_{e}}{dt}\right|}_{\mathrm{pk}}$	750.12	563.03	727.39	620.73
$C_{5{}^{\circ}}$	98.67	99.89	99.55	100
$L_{5{}^{\circ}}$	92	4	20	0

**Table 5:** Summary of displacement–error coupling (median values across sliding windows). Interquartile ranges (IQRs) for reference: $\rho_{x}\;\in \;\left[-0.083,\;0.179\right]$, $\rho_{y}\;\in \;\left[-0.156,\;0.136\right]$; ${\mathrm{lag}}_{x}\;\in \;\left[-100,\;107\right]$ ms, ${\mathrm{lag}}_{y}\;\in \;\left[-112,\;71\right]$ ms.

Metric (Median)	*x* Axis	*y* Axis
Correlation ρ [–]	0.025	−0.00265
Lag at max corr. [ms]	−16	+4

## Data Availability

The original contributions presented in this study are included in the article. Further inquiries can be directed to the corresponding author.

## Abbreviations

The following abbreviations are used in this manuscript:

BLDC	Brushless Direct Current Motor
CL	Closed Loop
CPU	Central Processing Unit
EKF	Extended Kalman Filter
MAE	Mean Absolute Error
MLP	Multilayer Perceptron
MSE	Mean Squared Error
NARX/NARXnet	Nonlinear Autoregressive Network with Exogenous Input
PMSM	Permanent Magnet Synchronous Motor
PLL	Phase-Locked Loop
P95	95th-Percentile Error
PSD	Power Spectral Density
PWM	Pulse-Width Modulation
RMSE	Root Mean Square Error
RPM	Revolutions Per Minute
SMO	Sliding-Mode Observer
